# The Role of Iron in Brain Development: A Systematic Review

**DOI:** 10.3390/nu12072001

**Published:** 2020-07-05

**Authors:** Samantha McCann, Marta Perapoch Amadó, Sophie E. Moore

**Affiliations:** 1 Department of Women and Children’s Health, King’s College London, London SE1 7EH, UK; sophie.moore@kcl.ac.uk; 2 Medical Research Council Unit, The Gambia at the London School of Hygiene and Tropical Medicine, Fajara, P.O. Box 273, Banjul, The Gambia; 3 Department of Medical Physics and Biomedical Engineering, University College London, London WC1E 6BT, UK; mp901@cam.ac.uk

**Keywords:** iron deficiency, brain development, pregnancy, infancy, pre-school, cognitive, motor, socio-emotional

## Abstract

One-third of children falter in cognitive development by pre-school age. Iron plays an important role in many neurodevelopmental processes, and animal studies suggest that iron sufficiency in pregnancy and infancy is particularly important for neurodevelopment. However, it is not clear whether iron deficiency directly impacts developmental outcomes, and, if so, whether impact differs by timing of exposure or developmental domain. We searched four databases for studies on iron deficiency or iron supplementation in pregnancy, or at 0–6 months, 6–24 months, or 2–4 years of age. All studies included neurodevelopmental assessments in infants or children up to 4 years old. We then qualitatively synthesized the literature. There was no clear relationship between iron status and developmental outcomes across any of the time windows or domains included. We identified a large quantity of low-quality studies, significant heterogeneity in study design and a lack of research focused on pregnancy and early infancy. In summary, despite good mechanistic evidence for the role of iron in brain development, evidence for the impact of iron deficiency or iron supplementation on early development is inconsistent. Further high-quality research is needed, particularly within pregnancy and early infancy, which has previously been neglected.

## 1. Introduction

Globally, one-third of children do not reach their developmental potential by pre-school age [1]. Children growing up in low- and middle-income countries (LMICs) are most at risk, with the burden of cognitive deficit lying disproportionately within sub-Saharan Africa [1]. The first 1000 days of life, from conception to two years of age, are widely recognised as the most important period for brain development. Deficits accrued within this period can have long-term consequences, including poorer academic achievement, mental health and economic productivity across the lifespan [2]. At the population level, these factors contribute to the continuation of the poverty cycle and stall progress. 

It is, therefore, crucial to understand why some children falter in their development and design preventative and corrective interventions accordingly. It is widely agreed that three broad factors are most influential: (i) infection and inflammatory stress, (ii) quality of care and (iii) nutrition [3]. The impact of these factors is likely additive, and they often co-occur, particularly in impoverished settings. As a potentially modifiable risk factor, a clear understanding of the role of micronutrient status, particularly iron, in early brain development is critical. 

Iron deficiency is the most common micronutrient deficiency worldwide and is particularly prevalent among pregnant women, infants and young children due to high iron demands during periods of rapid growth [4]. Iron deficiency progresses in stages, usually as a result of inadequate dietary intake, compromised absorption, which may be due to inflammation, or blood loss [5]. Initially, if iron supply does not meet demand, iron stores are used faster than they can be replenished, leading to iron depletion. Biochemically, this is characterised by a reduction in ferritin concentration, while measures of circulating iron (serum iron, soluble transferrin receptor (sTfR) and transferrin saturation (TSAT)) and red blood cell indices (mean corpuscular volume (MCV), mean corpuscular haemoglobin (MCH), zinc protoporphyrin (ZPP) and free erythrocyte protoporphyrin (FEP)) remain within the healthy range. Without intervention, iron depletion may progress to iron deficiency (ID), in which the body has insufficient iron to meet its current demands for normal function. This stage is biochemically indicated by a drop in serum iron and TSAT as well as an increase in sTfR. Iron regulation is adjusted to increase absorption and some iron-dependent activities are downregulated, as iron is preferentially used in red blood cell synthesis. If the iron deficit continues, ID progresses to iron deficiency anaemia (IDA), at which point red blood cell synthesis is compromised, characterised by reduced haemoglobin (Hb) concentration and further changes to the biomarkers of ID, mentioned above [6].

There is good mechanistic evidence from animal models and in vitro studies that iron is essential for many processes of brain development. Iron is an integral component of cytochrome C oxidase, the last enzyme in the oxidative phosphorylation pathway, and thus it is an essential component of intracellular metabolism. ID may, therefore, compromise the metabolically demanding processes involved in brain development [7]. Early ID may also lead to long-term downregulation of metabolic activity, due to changes in gene regulation following signalling cascades through mTOR, BDNF and MAP2 [8]. Evidence from animal models suggests that the hippocampus [9], the brain’s learning and memory hub, and the process of myelination, in which brain cells are insulated to increase the speed of neural processing [10,11], may be particularly vulnerable to ID. Iron is also essential in enzymes involved in monoamine (dopamine, adrenaline, noradrenaline and serotonin) production, meaning that socio-emotional development, executive function and memory processes reliant on these neurotransmitters may also be impacted by ID [12]. 

Despite this, evidence from human studies on the role of iron in brain development is equivocal. Many observational studies have identified a strong association between ID and cognitive outcomes [12]. However, this relationship may be confounded by other related factors such as co-occurring nutritional deficiencies, socio-economic deprivation, illness or poor caregiving. Iron supplementation trials have also had mixed results [13,14,15,16,17], suggesting that data from one setting may not be relevant for all contexts. Given the evidence from animal studies, we would expect that ID in pregnancy or early infancy would be more detrimental to neurodevelopment than if it occurred later, once the developmental foundations are laid [12]. Further, we may expect ID to differentially impact developmental domains, depending on the brain regions and processes developing most rapidly at the time of the deficiency [18,19]. However, neither of these hypotheses have been systematically investigated. Previous systematic reviews on this topic have either focused solely on specific developmental windows, such as pregnancy [13,14], infancy [15] or early childhood [16], or spanned wider windows of development and developmental domains without differentiating between them [13,17].

The aims of this review are to (i) investigate the relationship between iron status and brain development and (ii) assess whether this relationship differs according to age at exposure (pregnancy, 0–6 months, 6–24 months or 2–4 years of age) or developmental domain (neurophysiological function, cognitive, motor or socio-emotional development).

## 2. Materials and Methods 

We used the Preferred Reporting Items for Systematic Reviews and Meta-Analysis (PRISMA) guidelines in this review (Table S1) and registered the protocol with PROSPERO (Registration number: CRD42020171157).

### 2.1. Search Strategy and Inclusion Criteria 

Following search term development, searches were performed across four databases (Medline, EMBASE, Psych Info and Global Health) and studies published up to December 2019 were retrieved. All searches were filtered for texts written in English, but no other geographical limitations were set. 

Studies were included if they (i) measured iron status or administered iron supplementation, by any modality, to pregnant women and measured neurophysiological function, cognitive, socio-emotional, or motor skills in the offspring between birth and four years of age, or (ii) measured iron status or administered iron supplementation to infants or children up to four years of age and measured one or more of the aforementioned developmental outcomes up to four years of age. Observational studies were excluded if they did not compare cognitive outcomes among ID or IDA participants with non-ID/non-IDA controls. For example, studies comparing ID participants to IDA participants without an iron-sufficient (IS) control group were excluded, as they assess the impact of anaemia on brain development as opposed to ID, which is the focus of this study. Studies comparing anaemic participants to non-anaemic participants, where anaemia was not confirmed to be IDA, were excluded on the same basis. Studies measuring or supplementing iron in non-pregnant women and other age groups were excluded, as well as those with other outcomes (e.g., child growth). Randomised controlled trials (RCTs) where the effects of iron alone could not be separated from the effects of other micronutrients, macronutrients or other interventions were excluded. For example, studies of iron folic acid versus placebo or multiple micronutrients (MMNs) versus placebo were excluded, whereas studies of iron folic acid versus folic acid, or iron + MMNs versus MMNs were included. Studies of sickle cell anaemia and of anaemia without measures of ID were excluded, along with studies in other patient groups, e.g., diabetic mothers or very pre-term infants. Furthermore, studies were excluded if the outcomes measured did not fit into the pre-determined categories mentioned above, e.g., studies of infant sleep patterns. The main aim of the review was to assess the impact of iron supplementation and/or deficiency during different developmental windows (pregnancy, 0–6 months, 6–24 months, and 2–4 years). Therefore, studies including, but not differentiating between, participants spanning more than two of these windows (e.g., recruiting participants aged 6–59 months) were excluded. Studies spanning two exposure windows (e.g., 0–12 months) were included in the earlier window. In addition, reviews, reports, conference proceedings, letters, protocols and case studies were excluded. 

Search terms included Medical Subject Heading Terms [Anaemia, Iron Deficiency] AND [Pregnancy] OR [Child] OR [Infant] OR [New-born] AND [Child Development] OR [Cognition] as well as free text title and abstract searches for words related to pregnancy/infancy/early-childhood, iron deficiency and cognition. Full details of search terms are in Table S2. 

### 2.2. Study Selection and Data Extraction

Two researchers (S.M. and M.P.A.) independently screened titles and abstracts of all articles retrieved in the initial search. The full text of any article passing initial screening by at least one researcher was examined thoroughly to determine eligibility for inclusion. The data extracted from each eligible text was based on the criteria within the Cochrane Handbook [20]. The title, authors, date of publication, setting (country and LMIC/high-income country (HIC) status), study design, sample size, statistical methods, timing of iron measurements, methodology used to measure iron status, criteria for ID/IDA, prevalence of ID/IDA within the sample, comparison groups, timing and description of outcome measures were extracted from all articles, where possible. In addition, details about the duration, composition and administration of supplements, as well as the impact of supplementation on iron status were collected from all intervention studies, where reported. Articles reporting separate outcomes from the same initial study were identified and grouped together to prevent bias towards studies with multiple publications.

### 2.3. Data Quality Assessment 

The quality of each study was assessed using criteria based on Critical Appraisal Skills Programme (CASP) checklists [21], particularly focused on the design of the study, sample size and analytic approach. 

### 2.4. Synthesis of Results 

Due to the highly varied nature of the studies included within this review, a meta-analysis was not feasible. Instead, studies were divided into (i) observational (cross-sectional/longitudinal) or (ii) intervention (RCTs and other study designs where the exposure was iron supplementation), and then grouped according to the timing of exposure and developmental domain assessed. Articles reporting on intervention studies but using iron status as the exposure (i.e., not reporting by trial arms) were categorized as observational. 

## 3. Results

### 3.1. Description of the Literature

**Figure 1:**
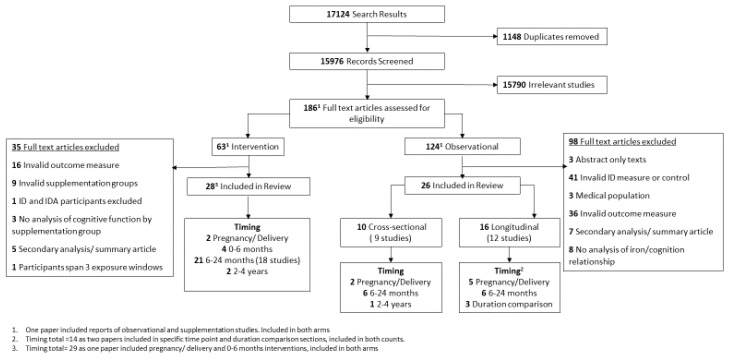
PRISMA flowchart of studies included in the review.

Of the 17,124 search results extracted from four databases, 1148 duplicates were removed, leaving 15,976 records to be screened by title and abstract. Screening identified 15,790 irrelevant articles and the full text of the remaining 186 articles were assessed for eligibility. Of these, 123 were observational studies of iron deficiency, 62 were iron supplementation studies and one text included both observational and intervention studies, so was included in both groups (Figure 1).

#### 3.1.1. Observational Studies

Following full text assessment, 26 observational articles were retained. The remaining 98 articles were excluded, largely due to inadequate measurement of ID (*n* = 41), e.g., by comparing anaemic participants to non-anaemic participants or inadequate cognitive outcomes (*n* = 36) such as parent-reported infant sleep patterns. The remaining 21 articles were excluded for other reasons, detailed in Figure 1. Of the 26 retained, 10 articles reported on nine cross-sectional studies, of which two focused on ID in pregnancy or at delivery, six at 6–24 months of age and one at 2–4 years of age. The remaining 16 articles reported 12 longitudinal studies; five focused on exposure in pregnancy or at delivery and six studies focused on postnatal exposure between 6 and 24 months of age. Three studies compared the impact of ID duration within different periods and are reported separately. 

#### 3.1.2. Intervention Studies

A total of 28 articles reporting on 25 distinct intervention studies met the full text inclusion criteria. A further 35 were excluded, mostly due to an invalid outcome measure (*n* = 16) or intervention (*n* = 9). A single study reported supplementation in pregnancy, three studies reported on infant supplementation between 0 and 6 months of age, 18 studies between 6 and 24 months, and two studies between 2 and 4 years of age. Finally, one study included arms supplementing pregnant women and/or their infants and was, therefore, included in both relevant sections (Figure 1). 

### 3.2. Summary of the Evidence

#### 3.2.1. Setting

Of the observational studies, 13 were conducted within LMICs—in Asia (*n* = 7), Central/South America (*n*= 3) and Africa (*n* = 3)—and seven were conducted in HICs (North America *n* = 6 and Europe *n* = 1). A single observational study spanned three sites across both LMICs and HICs (Ghana, China and the USA) (Tables S3 and S4). Of the 25 intervention studies, 14 were based in LMICs within Central and South America (*n* = 7) or Asia (*n* = 7), 10 were based in HICs (North America *n* = 5, Europe *n* = 4, Australia *n* = 1) and the location of one was not reported (Table S5).

#### 3.2.2. Exposures

There was considerable variation in the definition and measurement of ID/IDA among observational studies. Of those assessing the impact of ID in pregnancy/in utero, two studies measured maternal ID, one measured maternal IDA, three measured ID in cord blood at delivery and one study measured ID in both maternal and cord blood. Cord blood ID was defined as either ferritin <75 μg/L [22,23,24,25] or zinc protopophyrin (ZPP) >188 μmol/mol [26]. There was no consistency in the definition of maternal ID, although all studies measured ferritin concentration with [27,28] or without [24,29] a measure of inflammation. 

Among the studies assessing the impact of postnatal ID or IDA, six studies grouped participants based on whether they were IDA, ID or IS [30,31,32,33,34,35], five studies compared IDA participants to IS controls [36,37,38,39,40] and two studies compared ID to IS [41,42]. Other than four studies that used a single biomarker (ferritin, [34,36], sTfR [38], or MCV [37]), multiple biomarkers were used to define ID (including MCH, ZPP, sTfR and TSAT). The most common biomarkers were ferritin and MCV. However, there was substantial variation in the combination of biomarkers and some variation in the cut off values (Tables S3 and S4).

In a study in Ethiopia, Gashu et al. divided pre-school-aged participants into IDA, ID and IS groups based on measures of ferritin, Hb and alpha-1 acid glycoprotein (AGP) (IDA = ferritin <12 or <30 µg/L if AGP > 1.2 g/L and Hb < 11 g/dL; ID = ferritin <12 or <30 µg/L if AGP > 1.2 g/L; IS = ferritin ≥12 or ≥30 µg/L if AGP < 1.2 g/L and Hb ≥ 11 g/dL) [35]. 

#### 3.2.3. Outcomes

Direct observation of infant/child behaviour was the most common mode of developmental assessment, used in 16/26 observational studies and 20/29 supplementation studies (Figure 1). Behavioural assessment tools included the Bayley Scales of Infant and Toddler Development (BSID) [43], the Mullen Scales of Early Learning (MSEL) [44] and the Wechsler Pre-school and Primary Scale of Intelligence (WPPSI) [45]. Several studies (observational (*n* = 8), supplementation (*n* = 6)) used objective measures of brain structure or function, including assessments of visual or auditory physiological responses [23,24,25,36,40,46,47], structural magnetic resonance imaging (MRI) [29], eye-blink rates [48] and nerve conduction velocity [49]. Other methods of assessment included parent-reported behaviours, for example achieved motor milestones [50], language production [37] or problematic behaviours [37,51]. 

#### 3.2.4. Study Quality

Overall, there was a high risk of bias among the observational studies (Tables S3 and S4). Only three longitudinal studies [27,28,52] and one cross-sectional study [38] were determined to be at low risk of bias. None of the observational studies provided a sample size calculation to justify the number of participants, and many had relatively small sample sizes, with *n* < 30 ID or IDA participants in the analysis (Tables S3 and S4). Consideration of factors widely reported to be related to both developmental outcomes and ID, such as status of other micronutrients, measures of growth and socio-economic context was generally inadequate, and in many studies entirely absent from analysis, with only crude associations reported [25,29,32,34,37,42,53] (Tables S1 and S2). 

The quality of the intervention studies included in this review was also mixed; four RCTs were graded as having a low risk of bias [51,54,55,56], whereas 12 studies were determined to be at high risk of bias mainly due to small sample size, poor study design (including a lack of randomisation or blinding) or inadequate recognition of other nutritional deficiencies, socio-economic factors and quality of care, similar to the observational studies (Table S6). 

### 3.3. Iron Deficiency and Brain Development

#### 3.3.1. Observational Studies: Pregnancy 

##### Cognitive Development 

From a large (*n* = 828), well-powered longitudinal study in Benin, Mireku et al. reported no difference in the MSEL Early Learning Composite (ELC) scores at 12 months of age between infants born to mothers who were ID in pregnancy versus those who were IS [28]. However, a small Chinese study (ID = 35, IS = 92) found that infants born IS (cord blood ZPP/Heme ≤ 118 μmol/mol) displayed a typical recognition memory response at two months of age, whereas those born with ID (ZPP/Heme > 118 μmol/mol) did not [26,57]. 

##### Motor Development 

The study in Benin, from Mireku et al., failed to identify a relationship between maternal iron status (ID vs. IS) and infant motor development at 12 months of age [28]. Another large study (*n* = 523) in Vietnam reported that maternal ID in pregnancy was not associated with motor skills at six months of age [27]. However, it was noted that motor scores were lower among infants born to mothers with anaemia in late pregnancy, which was in turn associated with ID earlier in pregnancy [27]. 

##### Neurophysiological Development 

Neurophysiological assessments were used in four studies [23,24,25,29]. All four studies reported positive associations between iron status and infant responses. However, these results should be interpreted with caution as none of the studies were of high quality (Tables S3 and S4).

#### 3.3.2. Observational Studies: 6–24 Months of Age

##### Cognitive Development

A total of six longitudinal studies reported how cognitive performance differed with iron status within this developmental window. Fuglestad et al., in a study conducted among national and international adoptees in the USA (IDA = 8, IS = 55), found lower scores within the IDA group (Hb < 11 g/dL and two or more abnormal iron markers) (Table S4) compared to IS [40]. Using the same criteria for IDA, Doom et al. reported lower scores in both IDA and ID (Hb ≥ 11 g/dL and two abnormal iron markers) adoptees within the USA, compared to those who were IS [33]. However, in a study in Chile, the ID group performed largely in line with the IS group, with only the IDA group achieving significantly lower scores [32,53]. The results of a further two studies in the USA were inconclusive, with some, but unconvincing, differences between iron groups [39,41], whereas Beltran-Navarro et al. reported no difference in cognitive performance between IDA, ID and IS (IDA = Hb < 11 g/dL and ferritin < 12 µg/L, ID = Hb ≥ 11 g/dL and ferritin < 12 µg/L, IS = Hb ≥ 11 g/dL and ferritin ≥ 12 µg/L) groups within their study in Mexico [34]. Three further cross-sectional studies, conducted in populations from Bangladesh, France and the USA, reported no difference between cognitive outcomes among IDA, ID or IS participants [37,38,42]. 

##### Motor Development

A single longitudinal study, conducted in Chile, assessed motor development following postnatal ID in the first two years of life [32]. In this study, both IDA (Hb < 11 g/dL and two abnormal iron markers) and ID (Hb ≥ 11 g/dL and two abnormal iron markers) participants performed, on average, worse than IS participants, with the IDA group most severely affected. An additional three cross-sectional studies from Bangladesh (*n* = 434) [38], the USA (*n* = 106) [30] and a multi-centre study in Ghana, the USA and China (*n* = 209) [54] reported on motor outcomes. The study conducted in the US and the multi-centre study found some, but unconvincing, evidence of a motor deficit among IDA and ID participants compared to IS controls [30,54,58], and the third (Bangladesh) showed no difference between groups [38]. 

##### Socio-Emotional Development

The aforementioned USA-based longitudinal studies from Fuglestad et al. [41] and Carter et al. [39] also included measures of socio-emotional development and found evidence of a difference between some aspects of behaviour, but no consistent differences in scores based on iron status. A cross-sectional study in France [37] found no difference between IDA and IS participants. All three studies had very few ID and IDA participants (*n* < 30).

##### Neurophysiological Development

One small cross-sectional study (IDA = 25, IS = 25) conducted in India and a small longitudinal study in the USA assessed neurophysiological response to a visually evoked potential assessment and reported significantly longer peak and interpeak latencies among IDA participants compared to IS controls [36,40].

#### 3.3.3. Observational Studies: 2–4 Years of Age

Only one observational study focused on iron status from 2 to 4 years of age was identified within this review. The large study based in Ethiopia (*n* = 541), within which IDA was observed in 5.3% participants, compared cognitive scores measured by the WPPSI in pre-school-aged children (54–60 months of age), but found no association with ID or IDA (IDA = Hb < 11 g/dL and ferritin < 12 or <30 µg/L if AGP > 1.2 g/L; ID = serum ferritin <12 or <30 µg/L if AGP > 1.2 g/L) [35]. 

#### 3.3.4. Observational Studies: Duration Comparison

One study based in Mexico (*n* = 58) [34] along with two in China (*n* = 80 [57], *n* = 1194 [52]), took a different analytic approach, comparing the impact of timing or duration of ID on developmental outcomes. The Mexican study reported no difference in cognitive scores, but a significant deficit in motor and language performance at 14–18 months of age among a group of infants with chronic ID (ferritin < 12 µg/L at both six and 14–18 month of age) compared to those who were IS at both six and 14–18 months of age [34]. In China, Santos et al. reported that infants with either prenatal (cord ferritin  < 75 µg/L or ZPP/Heme > 118 µmol/mol) or postnatal ID (body iron < 0 mg/kg) performed worse on gross motor tasks at nine months of age, than infants who were IS in either or both periods [52]. However, ID in both periods had no greater impact on performance than ID in a single period. Armony-Sivan et al. reported from a separate Chinese study that there was no difference in overall response to emotional stimuli among the prenatal ID (cord blood ferritin < 75 μg/L or ZPP/Heme > 118 μmol/mol), postnatal ID (MCV < 74 fl, red cell distribution width (RDW) > 14.5%, ferritin < 12 μg/L, ZPP/H > 69 μmol/mol heme at 9 months of age), chronic ID (both pre- and postnatal ID) and never ID groups when assessed by electroencephalography (EEG) at 9 months of age, although the chronic ID group had a different topographical response pattern compared to the other groups [57]. 

#### 3.3.5. Intervention Studies: Pregnancy

The impact of iron supplementation administered to women during pregnancy on developmental outcomes in their offspring was reported in two studies. Firstly, a four-arm RCT in China administered iron folic acid (30 mg/d iron + 400 µ/d) or folic acid alone (400 µg/d) from <20 weeks gestation to delivery [59], and further randomised infants from six weeks to nine months of age with oral iron (1 mg/kg/d) or placebo [54]. Secondly, in an RCT among pregnant women in Australia, participants similarly received a low dose of iron (20 mg/d) or placebo tablet daily during the second half of pregnancy [51]. Both trials were deemed to have a low risk of bias. 

##### Cognitive Development

Zhou et al., in their trial in Australia, found no evidence of a difference in cognitive scores between the children of iron-supplemented or control women at four years of age [51].

##### Socio-Emotional Development

In the same Australian trial, there was no difference in mean socio-emotional scores between trial arms. However, a higher proportion of the iron-supplemented group scored within the ‘abnormal’ range on a parent-reported measure of childhood behavioural problems at four years of age [51]. 

##### Motor Development

In the Chinese study, no effect was observed of antenatal iron supplementation on offspring motor development at nine months of age [54]. 

#### 3.3.6. Intervention Studies: 0–6 Months of Age

In total, three double-blind RCTs administering iron supplements in early infancy were identified. Firstly, as described above, the trial in China from Angulo-Barroso et al. included a postnatal arm, with infants randomised to receive iron or placebo supplementation from six weeks to nine months of age [54]. In Canada, Friel et al. reported on a small trial (*n* = 77) supplementing infants with iron (7.5 mg/d) or placebo syrup from 1 to 6 months of age [60]. Moffatt et al. reported a larger Canadian trial (*n* = 283) comparing the impact of feeding infants ad libitum with iron-enriched (12.8 mg/L iron) versus regular formula (1.1 mg/L iron) [55]. In addition, Otero et al. compared cognitive performance among a small group of IDA infants (*n* = 50) aged between 3 and 12 months in Mexico, before and after daily iron supplementation (5 mg/kg/d) for four months [61]. All three RCTs were rated at either low or medium risk of bias. The Mexican study was determined to be at high risk of bias, given its small size and lack of control group. 

##### Cognitive Development

Moffatt et al. and Friel et al. used the BSID mental development index (MDI) to assess the impact of iron supplementation on cognitive function but found no relationship between supplementation during early infancy and developmental scores in toddlerhood [55,60]. 

##### Socio-Emotional Development

Only one of the trials, from Canada, assessed infant socio-emotional development but found no difference between groups [55]. 

##### Motor Development

All three RCTs measuring motor outcomes in infants and toddlers found improved scores among supplemented infants compared to controls. In China, Angulo-Barroso et al. observed that infants in the iron supplementation group achieved higher gross motor scores than infants administered placebo in infancy, when assessed by the Peabody Developmental Motor Scale (PDMS-2) [62] at nine months of age (*d* = 0.16) [54]. Both Canadian trials used the BSID psychomotor development index (PDI) to assess infant motor skills at 12 months of age and found a similar difference in mean scores between the groups, with iron-supplemented infants scoring on average seven points higher (100 +/−12 vs. 93 +/−8.8) [60] and 6.3 points higher (100.5 +/−14.1 vs. 94.2 +/−12.6) than the control group [55]. Moffatt et al. also assessed motor skills by the BSID PDI at 18 months, but the difference between supplementation and placebo groups was no longer significant, despite the maintenance of improved iron parameters within the supplemented group [55]. 

##### Neurophysiological Development

In the Mexican study assessing developmental outcomes before and after three months of daily iron supplementation (5 mg/kg/d), Otero et al. compared neurophysiological responses using quantitative EEG and found that IDA infant responses were abnormal prior to, and normalised following, the period of supplementation [61]. 

#### 3.3.7. Intervention Studies: 6–24 Months of Age

A total of 18 studies focusing on supplementation in infants aged 6–24 months met the inclusion criteria for this review. Of these, 12 were located in LMICs, almost half of which were in the Americas (Chile *n* = 3, Costa Rica *n* = 2, Guatemala *n* = 1, Turkey *n* = 3, Indonesia *n* = 2, and Bangladesh *n* = 1), and six were conducted in HICs (the USA *n* = 3, the UK *n* = 2, and Spain *n* = 1). Just over half of the studies were RCTs (10/18), but only a single study was determined to be at low risk of bias [56]. The remaining studies compared developmental scores before and after supplementation and were largely low in quality. Supplementation was administered either via injection or orally through drops, syrup or infant formula. Duration of oral supplementation ranged from 7–10 days to nine months and there was considerable variation in the dosing of supplementation regimens across studies. Some studies dosed participants individually based on their weight [47,63,64,65,66] (doses ranging from 3 to 6 mg/kg iron per day) or by iron status of the participant at baseline [67]. Others supplemented all participants with the same dose of iron regardless of age, infant weight or iron status [48,56,68,69]. In studies using infant formula as the mode of iron supplementation [50,70,71], infants received formula ad libitum, so iron dose varied between participants based on consumption and composition of formula (which ranged from 1.2 to 12.7 mg/L iron). All studies conducted developmental assessments within a week of completing the intervention period. In addition, the studies in Costa Rica and Chile reported by Lozoff [72] and Walter [32], respectively, conducted developmental assessments at an interim point during the supplementation period. There was substantial variation between studies in the age of enrolment into trials. Some studies recruited participants within a very narrow age range of 1–2 months [32,48,50,56,68,69,70,71], whereas other studies recruited more broadly. For example, the study in Guatemala [63] and two studies in Turkey [46,47] recruited participants from 6 to 24 months of age.

##### Cognitive Development

A total of 14 studies assessed cognitive performance in relation to supplementation within this developmental window. The BSID was the most common tool of assessment, used in all 14 studies. In addition, Akman et al. [66] used the Denver Developmental Screening Test (DDST) [73] and Lozoff et al. [50] used the Fagan Test of Infant Intelligence [74]. Of the 14 studies, six reported improved scores among the supplemented group [65,66,67,72,75,76], three of which were deemed to be at medium risk of bias and three at high risk. In all six studies, the effect was seen, either solely or more strongly, in participants who were IDA at baseline. Further, one study in Costa Rica found that infants within the supplemented group who became IS scored better when assessed by the BSID MDI at the study end line, although there was no difference between the scores of the supplemented and placebo groups overall [64]. In the remaining seven studies, there was no difference in the BSID MDI between intervention arms [32,50,56,63,68,70,71]. 

##### Motor Development

Fifteen studies assessed the impact of iron supplementation between 6 and 24 months of age on motor development. Aukett et al. assessed motor skills using the DDST [73] and found no difference between the mean scores of supplemented and control group. However, a higher proportion of infants in the iron group reached the ‘expected developmental level’ by end line [69]. The remaining 14 studies used the BSID PDI as the assessment tool, two of which reported improved motor scores following iron supplementation [56,66] and a further two studies reported a benefit of iron supplementation on a subgroup of participants; those who were IDA at baseline [65], or those who became IS by end line [64]. Nine studies reported no difference in the BSID PDI scores between groups (Table S5) and one study did not report results for this measure, despite including the BSID PDI within the methodology section of their paper [76]. 

##### Socio-Emotional Development

Within seven studies, the behavioural component of the BSID (IBR (infant behaviour record) or BRS (behaviour rating scale)) was used to assess socio-emotional development. One study found no difference between supplementation groups [56], four studies identified differences between supplementation groups in some assessed items, but no consistent differences [50,68,72,75] and two studies did not report results for this measure [67,76,77]. 

##### Neurophysiological Development

Two small supplementation studies conducted in Turkey (IDA *n* = 25, ID *n* = 24, and IS *n* = 44) [47], (IDA *n* = 20 and IS *n* = 20) [46] used physiological measures of neurodevelopment. Neither study identified a difference in response among IDA infants compared to non-IDA controls at baseline, or a difference between supplemented and non-supplemented participants at end line. However, Lozoff et al., in a study conducted in the USA, identified a difference in eye-blink rates in IDA (*n* = 19) and non-IDA (*n* = 42) infants at baseline, which was corrected by iron supplementation [48]. 

#### 3.3.8. Intervention Studies: 2–4 Years of Age

Only two studies administering iron supplements to young children aged 2–4 years were identified. Metallinos-Katsaras et al. conducted an RCT in IDA pre-schoolers (36–59 months of age) and non-IDA controls in Greece [78]. Although little difference was identified in the performance of IDA and non-IDA children at baseline, iron-supplemented IDA children performed better than those treated with an iron-free MMN at end line (IDA *n* = 21 (14 iron and 7 iron-free MMNs), non-IDA *n* = 28 (18 iron and 10 iron-free MMNs)) [78]. Kabakus et al. reported on a study, the location of which was not reported, among 30 young children (IDA *n* = 18 and non-IDA *n* = 12) aged 25–42 months. At baseline, slower nerve conduction velocity was observed among the IDA group. However, following supplementation (6 mg/kg/d) for 3 months, the IDA group performed in line with non-IDA baseline results (non-IDA were not reassessed at end line) [49]. Both studies were considered at high risk of bias (Table S5). 

## 4. Discussion

The main aim of this systematic review was to assess the relationship between iron status or iron supplementation and brain development, across specific age windows within the first four years of life (pregnancy, 0–6 months, 6–24 months, and 2–4 years). A total of 54 relevant papers were identified—26 from observational studies of iron status and 28 from iron intervention studies. However, despite the relatively large number of studies, the evidence was equivocal. Within the included literature, observational studies varied substantially in the assessment and definition of ID. Similarly, intervention studies varied in the dose, duration and mode of supplementation. Further, a diverse range of assessment tools and techniques were used to measure developmental outcomes. This considerable heterogeneity between studies prohibited a meta-analysis. Nonetheless, several interesting themes emerged which are discussed qualitatively below. 

### 4.1. Windows of Exposure

#### 4.1.1. Pregnancy

Seven studies included in this review assessed the relationship between maternal or foetal (cord blood) ID and developmental outcomes, which were measured between birth and 12 months of age. The majority of the studies (5/7) identified some evidence that prenatal ID was detrimental to early brain development. Four of the five studies showing impact used neurophysiological assessments as an outcome measure, including structural MRI and auditory brainstem response, whereas the two studies which found no impact assessed cognitive and motor development using behavioural assessments (the BSID). Emerging evidence from research into autism spectrum disorder suggests atypical neural responses are identifiable from 4 to 6 months of age, significantly prior to observable changes to behaviour [79]. Given that all studies measured developmental outcomes in the first year of life, it is possible that in this age group, neurophysiological tools were able to identify iron-related neural differences which had not yet manifested behaviourally. However, the studies using physiological tools were overall of lower quality than those using behavioural assessments, and therefore differences in findings may be due to bias.

Disparity in the study findings may have also been due to differences in comparison groups. Four of the five studies that reported an association between iron status and developmental outcomes compared infants who were ID or IS (measured from cord blood at delivery), and the final study compared infants born to IDA versus IS mothers. The two studies that reported no association between iron status in pregnancy and developmental outcomes compared infants born to mothers who were ID versus IS. Evidence suggests that upregulation of maternal–foetal iron transfer ensures that foetal iron accumulation is normal in cases of mild–moderate maternal ID [80,81], whereas maternal IDA is likely to result in foetal ID [82,83]. This means that infants in the ‘ID’ group of the two studies comparing maternal ID versus IS may have been iron replete, which may in part explain the lack of difference in developmental outcomes between iron status groups in these studies. Despite the lack of publications focused on this developmental window, there is mounting evidence that foetal ID is detrimental to neurodevelopment. However, the low quality of available evidence may undermine this message. High-quality research focused on foetal ID, as opposed to maternal ID, and early development is needed to substantiate this area of research and eliminate doubts regarding the reliability of findings.

Only two studies reported the impact of iron supplementation in pregnancy on developmental outcomes in infancy. Although both were high-quality RCTs with a low risk of bias, neither study found any developmental impact. However, one of the two studies was conducted among women at low risk of IDA [51], and therefore, although the impact on infant iron status was not reported, supplementation was likely of little benefit [84]. The second study specifically reported no impact of maternal iron supplementation on the iron status of infants at delivery (cord blood ferritin) [54]. The negative results of these two trials could, therefore, be misinterpreted as a lack of impact, when in fact they represent the lack of appropriate evidence. High-quality research into the impact of iron supplementation in pregnancy on developmental outcomes is needed. This research should be conducted in populations where ID/IDA is common and include measurement of infant iron status to determine the efficacy of the intervention in maintaining infant iron sufficiency. In addition, given the limited efficacy of maternal iron supplementation in the prevention of postnatal ID in previous trials [54,85], further research into interventions that more effectively maintain iron sufficiency in utero and throughout infancy would be beneficial.

#### 4.1.2. 0–6 Months

When looking at the period of early infancy (0–6 months of age), no papers reporting observational studies were identified, but four relevant studies reporting iron supplementation in the first six months of life were included in this review. Unlike the interventions in pregnancy, all four studies reported some improvement in iron status in the supplemented group. Further, all studies reported a benefit of iron supplementation on developmental outcomes, although these benefits were limited to specific domains. The three RCTs assessing motor development, which were graded as at low or medium risk of bias, reported enhanced motor performance at 9–12 months of age [54,55,60]. However, neither of the two studies assessing cognitive outcomes reported any difference between trial arms [55,60], nor did the single study assessing socio-emotional development [55]. This domain-specific impact of supplementation may reflect the relatively rapid development of motor skills in the first year of life [86]. Equally, failure to identify cognitive or socio-emotional differences may be due to the poor reliability and sensitivity of behavioural tools used in young infants. 

It is interesting that there are no studies assessing the impact of ID/IDA from 0 to 6 months of age. This may be due to the assumption that infants of this age, especially when breastfed, are at low risk of ID [87], which is supported by the exclusion of infants under six months of age from WHO recommendations of routine iron supplementation for children in regions of endemic IDA [88]. However, protection from ID in this age group, and especially in infants who are exclusively or predominantly breastfed, requires that (i) infants are born to mothers with adequate iron during pregnancy, (ii) they are not born premature or at low birthweight, and (iii) delayed cord clamping was practiced at delivery [87]. In settings where IDA during pregnancy is prevalent, it is rare for these conditions to all be met, placing young infants at risk of ID [89,90]. These findings reinforce the evidence that interventions to maintain iron sufficiency throughout foetal and early infant development, as opposed to correction of ID thereafter, are more likely to be effective in supporting early brain development. Although evidence is currently sparse, we have identified encouraging findings supporting the possible benefit of iron supplementation in early infancy on developmental outcomes. There is a need for further well-conducted studies within this previously neglected age group. 

#### 4.1.3. 6–24 Months

Of the studies included in this review, the most common window of investigation was 6–24 months of age. However, despite the inclusion of 26 studies in total, there were limited consistent findings across studies to support robust conclusions. A total of 12 observational studies were identified; seven reported a positive relationship between iron status and developmental outcomes. No relationships were observed in the other five studies. Unlike observations in the younger age group, the effect did not appear to be domain specific, with similar findings across cognitive, motor and neurophysiological assessments. There were no obvious similarities in setting, study design, measurement of exposure or outcome or analytic approach among studies with similar findings. However, it must be noted that the research was largely assessed as low quality, limiting further interpretation. 

Similarly, around half (10/18) of the intervention studies in the 6–24 month age group reported higher scores in developmental assessments following iron supplementation. Again, study quality was mixed, and eight studies were graded at high risk of bias. With a greater number of papers focused on this developmental window, we were able to separately consider the findings of the higher-quality studies alone, all of which were RCTs (9 at medium risk of bias and 1 at low risk of bias). Among these 10 studies, four identified a developmental benefit of iron supplementation and six found no benefit. Some interesting parallels were drawn between the studies with similar findings. Firstly, those reporting a benefit of supplementation were conducted among higher-risk populations (LMIC and/or high proportion of IDA participants at baseline). Further, all four studies that identified a difference between trial arms administered daily iron drops or syrup, at a dose of at least 10 mg iron per day and for at least 3 months, in line with the WHO recommendations for prevention of IDA in at-risk populations [88], and reported improved iron status among the supplemented group. In contrast, the studies that reported no difference between trial arms administered supplements which were of a lower dose (e.g., 20 mg per week [68]), a shorter duration (e.g., 7–10 days [63,91,92]) or, in the three studies using infant formula, less controlled and compared to a lower dose of iron as opposed to no iron. These interventions were less effective, with only half of the studies reporting a difference in iron status between trial arms [50,69,71]. These findings suggest that differences in study design, setting and formula of intervention contributed to differences in results. All studies, irrespective of findings, used similar behavioural assessments (the BSID or DDST), meaning that the mode of assessment is less likely to have impacted the results, although participants in the studies that identified impact were slightly older at time of developmental assessment (12–24 months of age) than those in the studies that reported no impact (6–19 months of age), which may have altered the reliability of the assessments. 

#### 4.1.4. 2–4 Years

Despite wide acknowledgement that the first 1000 days of life are the most important period for brain development, we included studies focused on ID or supplementation in two to four year olds, primarily to ascertain whether evidence corroborates theory that early developmental deficits from ID are more difficult to overcome once this window has passed. Unfortunately, our literature search identified only three relevant studies in this age window—a single observational study assessing the relationship between ID and school readiness and two intervention studies, assessing the impact of iron supplementation on neurodevelopmental outcomes within this age group, both of which had a high risk of bias. This lack of data precluded our ability to draw any reliable conclusions regarding the relationship between iron deficiency or supplementation and developmental outcomes within this age group. 

### 4.2. Developmental Domains 

#### 4.2.1. Motor Development

The impact of ID on motor development is likely through two main mechanisms: (i) altered basal ganglia function as a result of dopaminergic changes, and (ii) compromised myelination of the motor cortex and associated areas. Processes underlying both of these mechanisms occur predominantly in late gestation and early infancy [86]. We found some evidence that iron supplementation in early infancy (0–6 months of age) promotes motor development, whereas iron supplementation in later infancy or early childhood may be less beneficial. Our findings support the hypothesis of differential impact based on timing and suggest iron status up to six months of age may be particularly important for motor development. 

#### 4.2.2. Socio-Emotional Development

Given the robust evidence from animal studies [12], we expected to identify an association between ID and socio-emotional development. However, evidence of this relationship was not as strong as hypothesised. This may have been due to a lack of setting- and age-appropriate tools, which has previously been recognised as a limitation within the field [93]. Further, the most common form of socio-emotional assessment was examiner-reported behaviour during an assessment. However, this measure is neither specific to affect nor representative of naturalistic behaviour. Finally, there was no consistency in the reporting of socio-emotional outcomes across studies, even when the same tool was used. When multiple assessments were conducted, agreement was low, suggesting low reliability [39].

#### 4.2.3. Cognitive Development

Evidence relating iron status and cognitive development was equivocal. Given that iron is more important to some aspects of cognition than others, the lack of association may be a result of the broad assessments used in most studies (e.g., the BSID, the MSEL, and the WPPSI) which combine many aspects of cognition, such as memory, language, attention and fine motor skills, within a single total score. Studies that used specific tests based on mechanistic evidence, such as those related to speed of processing, memory or executive function [26,33,39], were able to identify a relationship between iron status and outcomes, which may suggest that they were more sensitive to iron-related neural changes. Further, given the extended period of cognitive development, it is possible that behavioural deficits associated with ID in early development become more apparent with age. Our findings may have been limited by the age cut off for outcome measures. 

### 4.3. Strengths and Limitations 

This review had several strengths. Firstly, the comprehensive approach, with literature drawn from four databases without geographical or date limitations, and including both observational studies of ID and studies of iron supplementation, has allowed us to consider a large range of relevant literature from different contexts and with varying study design. Secondly, we included studies assessing ID or supplementation across the first 1000 days of life, which is widely acknowledged as the most important window of development, but also looked more specifically at narrower windows of development, in an attempt to identify the period in which iron is most critical. Further, based on mechanistic evidence on the roles of iron in brain development, we included outcomes across a range of relevant developmental domains, some of which were absent from previous reviews [17]. 

Despite this robust methodology, the review was unable to find substantial, consistent evidence relating iron status and brain development, particularly that which focused on pregnancy, early infancy (0–6 months) or pre-school children (2–4 years). We also identified a considerable number of low-quality studies with a high risk of bias, the results of which must be interpreted with caution. 

In addition, collation of evidence and comparison of exposure windows was hindered by significant heterogeneity between studies. For observational studies, variation in setting, comparison groups (ID/IDA/IS), measurement and definition of ID/IDA, timing of outcome measures, developmental assessment tools and analytic approach was substantial. In addition, among intervention studies, the timing, duration, dosing and method of supplementation were also highly varied.

Many of the studies included in this review were conducted in settings where ID and IDA is prevalent, for example in LMICs (*n* = 29) or among vulnerable groups in HICs (low socio-economic status [37,39,94,95] or national and international adoptees [33,40,41]). However, the most vulnerable populations were underrepresented in the published literature. Sub-Saharan Africa disproportionality carries the burden of ID, yet not a single intervention study and only three observational studies [28,31,35] were conducted within this region. It is likely that the impact of ID and iron supplementation is dependent on wider factors such as infant morbidity or inflammation, food intake and social or cultural influences [96,97,98]. Therefore, it may not be possible to extrapolate findings from one setting to another, and efforts should be made to ensure future research is conducted within, and relevant to, the populations who could benefit most. 

The majority of studies identified through this review were heavily reliant on behavioural assessments to measure developmental outcome; however, such assessments are less sensitive to developmental deficits when used in infants under 12 months of age and may be less reliable outside the HIC settings in which they were developed [99,100,101]. From the data presented in the current review, neurophysiological tools appeared to offer greater sensitivity in detecting differences between groups. However, these were largely used in low-quality studies and, unlike standardised behavioural tests, which have been shown to reasonably predict important later outcomes [2,102,103,104], the longer-term relevance of differences identified by neurophysiological assessment are not yet fully understood. Further research to identify meaningful early indicators of developmental deficit, suitable for use in the most vulnerable settings and age groups, would be very beneficial to this field of research [105,106]. Of particular concern is the lack of tools for the assessment of socio-emotional development in infants and young children, especially given the strong animal evidence that socio-emotional development is compromised as a result of changes to dopaminergic neurotransmission in periods of ID [12]. Many studies used the BSID IBR or BRS for this purpose. However, for this scale, unlike the mental and motor scales, which can be converted into a single score, there was no standard way of reporting the IBR, which made results difficult to interpret [107].

Although iron deficiency is the most common micronutrient deficiency and iron has multiple roles in neurodevelopment, nutritional deficiencies often co-occur [108,109], raising the importance, in observational studies, of other micronutrient deficiencies on neurodevelopment [18]. Only one observational study included within this review measured other micronutrients alongside iron [35]. They reported that selenium deficiency and anaemia, but not ID alone, were associated with lower cognitive scores. The lack of a consistent relationship between iron status and outcome from the observational studies reviewed here is possibly a consequence of confounding by other micro- or macronutrient deficiencies. Similarly, micronutrient status was rarely measured within the intervention studies; it is possible that the impact of iron supplementation on development was dependent on the availability of other nutrients [110]. For example, although Black et al. reported no improvement in motor skills as a result of iron supplementation alone, infants in the same trial who received combined iron and zinc supplementation did show improvement [68]. More comprehensive nutritional profiling is needed to better understand these complex relationships.

Based on the evidence that as many as one-third of children are failing to reach their developmental milestones by the time they start school [1], we limited the scope of this review to studies where developmental outcomes were measured in children under 5 years of age. The rationale behind this was to (i) determine whether ID/IDA plays a role in early developmental deficits specifically, and (ii) to limit further heterogeneity between study outcomes, as assessments used in older children and adults such as educational attainment, IQ and mental health cannot be assessed in infants and young children. However, early brain development forms the foundations upon which subsequent developmental processes depend, and tools for developmental assessment of infants and young children remain limited. A number of recent publications have reported on the long-term impact of ID in infancy, even following correction before two years of age. These include delayed neural response [111,112] and physical reaction times [112] at 10 years of age, higher rates of internalising and externalising behavioural problems in adolescence [113], consistently lower scores in intelligence assessments up to 19 years of age [114], reduced neural connectivity at 22 years of age [115] and poorer educational achievement, interpersonal relationships and mental health at 25 years of age [116]. It is possible that the impact of early deficits become more pronounced, and more identifiable over time. Therefore, our limited age of outcome may have impacted the findings from this review.

Finally, this review focused on the impact of iron deficiency on developmental outcomes. However, we must also acknowledge the possibility of adverse effects of iron supplementation, Previous trials among iron-replete children have reported poorer cognitive outcomes in the iron-treated group, whereas trials among ID or IDA participants have not reported such adverse effects [117]. These findings emphasise the importance of conducting such trials in populations where ID/IDA is endemic, not only to maximise benefit but also to minimize risk. Iron supplementation has also been associated with increased risk of malaria in malaria-endemic regions. However, a recent systematic review concluded that supplementation in children (0–18 years) is effective in improving iron status and reducing anaemia, and does not pose increased risk as long as adequate malaria prevention and treatment services are implemented [118].

### 4.4. Future Directions

This review has highlighted the need for further research in this field. However, in order to make progress and use resources most effectively, future studies should address the issues that limited previous work.

Firstly, research should be based on mechanistic evidence. Animal studies clearly demonstrate that ID in utero and early infancy has long-term neural impact, particularly on myelination, learning and memory and basal ganglia function [86]. Guided by this evidence, future studies should use age-appropriate outcome measures focused on developmental domains of interest. For example, to address the impact of ID on the developing hippocampus and associated learning and memory functions, structural MRI could be used from birth to assess impact on physical hippocampal development and functional brain-imaging assessments such as EEG or fNIRS, alongside objective tools such as eye tracking, could be used to conduct learning and memory assessments without relying subjective assessment of infant behaviour. Further, measurement of ID should be standardised, and research should be conducted in areas with endemic ID/IDA. WHO guidelines released in 2020 recommend the use of ferritin as a marker of iron deficiency and to monitor the impact of iron supplementation in all age groups. In populations where inflammation or infection is widespread, ferritin should be measured concurrently with two acute phase response proteins such as AGP and CRP [119]. Finally, other contributing factors should be accounted for, particularly other nutritional deficiencies and exposures. In supplementation studies, the nutritional backdrop should be characterised as thoroughly as possible and contribute to trial design and evaluation. This is especially relevant in relation to (i) lead exposure, which can impact iron absorption [120] and has independent effects on brain development [121]; (ii) zinc deficiency, which is common in populations with endemic ID, can be exacerbated by iron supplementation [122], and may independently impact cognitive outcomes [123]; and (iii) copper deficiency, which may impair iron absorption [124] and impact cerebellar development [125]. Trial designs which draw on findings from previous studies on micronutrient interactions [122] and strategically incorporate combinations of micronutrients may be beneficial. In observational studies, measurement of zinc, lead and other micronutrients of interest should be implemented more widely and more advanced statistical techniques, including structural equation modelling, could be used to better understand the complexities of social, environmental and nutritional influences on the relationship between ID and brain development.

## 5. Conclusions

Given the established roles of iron in processes of neurodevelopment, it is likely that ID or IDA does impact cognitive development, particularly if it occurs in utero or early infancy. However, the strength of the current evidence remains equivocal and neither a threshold for impact nor critical period has been established within this review. This is likely due, at least in part, to the disparity between mechanistic evidence and translational research. Despite robust mechanistic evidence that (i) brain development is most rapid in pregnancy and early infancy and (ii) maintenance of iron sufficiency is preferable for neurodevelopment over correction of ID, human research has been focused on reversal of ID in the second year of life. Further, differences in study design and quality limited direct comparability between studies, and likely contributed to the disparity in findings.

Further high-quality studies on the impact of iron supplementation on developmental outcomes are needed. These studies should: (i) focus on maintenance of iron sufficiency in utero and early infancy, in line with mechanistic evidence; (ii) be conducted within populations who are at high risk of ID/IDA; (iii) take into account the possible impact of other factors including nutritional deficiencies; (vi) establish standardisation of ID and IDA definition and measurement across studies to aid comparison; (v) use tools that are capable of reliably measuring developmental outcomes in young infants across the range of developmental domains.

## Supplementary Materials

The following are available online at https://www.mdpi.com/2072-6643/12/7/2001/s1, Table S1. PRISMA Checklist; Table S2. Search Strategy; Table S3. Cross-Sectional Studies; Table S4. Longitudinal Studies; Table S5. Intervention Studies; Table S6. Risk of Bias.
