# Supplementary material for: The Role of Iron in Brain Development: A Systematic Review
Source: Nutrients. 2020 Jul 5;12(7):2001. doi: 10.3390/nu12072001 (PMC7400887; doi:10.3390/nu12072001)
Supplement: Supplementary file 1 [file nutrients-12-02001-s001.zip › Supplementary material/Tables S3-S6 McCann et al 2020.docx]

| **Table S3 Cross-sectional Studies** | | | | | | | |
| --- | --- | --- | --- | --- | --- | --- | --- |
| **Author (year)** | **Sample size** | **Country** | **Iron measurements** | **Comparison Groups (n)** | **Outcome Measure** | **Results** | **Risk of Bias** |
| **Pregnancy / Delivery** | | | | | | | |
| Basu et al.  (2018) [29] | 90 | India (LMIC) | **Maternal blood at delivery**  IDA =  Hb <11g/dL and Ferritin <12 μg/L  IS= Hb≥11 g/dL and Ferritin>12 μg/L | IDA (70)  IS (20) | **Physiological**  MRI scan for hippocampal volume | **Physiological**  IDA group had reduced hippocampal volume compared to IS group. | **High**  No adjustment for confounding. |
| Pallone et al.  (2018) [23] | 109 | Brazil (LMIC) | **Cord blood at delivery**  ID= Ferritin ≥11 and ≤75 μg/L)  IS= Ferritin >75 μg/L | ID (38)  IS (71) | **Physiological**  BERA hearing assessment | **Physiological**  Some longer wave latencies and inter-wave latencies in ID group | **Medium**  Some adjustment but relatively small sample and unknown relevance of difference in outcome. |
| **0-24 months** | | | | | | | |
| Angulo-Barroso et al. (2011) [31] | 209 | Ghana, China (LMIC) USA (HIC) | **9 months of age**  IDA= Hb < 11 g/dL, MCV <74 fl and/or RDW >14%  ID= Hb ≥ 11 g/dL, together with MCV < 74 fl and/or RDW > 14%.  IS= Hb ≥ 11 g/dL, MCV ≥ 74 fl, and RDW ≤ 14% | Ghana: IDA (55), ID (43), IS (2)  China: IDA (19), ID (35), IS (46)  USA: IDA (32), ID (29), IS (39) | **Motor**  Gross motor milestones and a range of behavioural fine motor tasks | **Motor**  No significant association between iron group and gross motor score when adjusted for site. IS infants performed significantly better on one of the five fine motor tasks. | **Medium**  Site differences difficult to overcome even when adjusted for. No specific analysis per site by iron status. |
| Delinard et al. (1981) [42] | 212 | USA (HIC) | **11-13 months**  Severely ID= ferritin <=9g/L  Mildly ID= ferritin >9 & <=20g/L  IS= Ferritin >20g/L | Severe ID (34)  Mild ID (21)  IS (157) | **Cognitive**  BSID MDI, habituation assessment, Uzginis and Hunt Ordinal Scales of Psychological Development | **Cognitive**  No difference in scores between groups. | **High**  Small sample size, no adjustment for confounding. |
| Honig et al.  (1993) [37] | 177 | France (HIC) | **8-14 months of age**  IDA =Hb<10.5 g/dL & MCV <73 fl  IS= Hb >=11.5g/dL and MCV>=73 fl | IDA (22)  IS (155) | **Cognitive**  Brunet-Lezine Development Test  **Socioemotional**  Parent-report of child behaviour difficulties  **Language**  Parent-report of language delay. | **Cognitive, Language, Socio-emotional**  No difference in scores between IDA and IS group | **High**  Small sample size, multiple comparisons, no adjustment for confounding. |
| Monga et al. (2010) [36] | 50 | India (LMIC) | **6-24 months of age**  IDA= Hb<10.5 g/dL MCV < 70 fl, MCH<23 pg, MCHC<30 g/dL), ferritin <12 μg/L, TSAT <10%  TAB  IS= Hb >=10.5g/dL, ferritin>12 μg/L and TSAT >10% | IDA (25)  IS (25) | **Physiological**  Visually evoked potential. | **Physiological**  Significantly longer peak latencies in IDA group. | **High**  Small sample size, no adjustment for confounding, unknown relevance of difference in outcome measure. |
| Shafir et al. (2008, 2009) [30, 58] | 2008 106  2009 9 | USA (HIC) | **9-10 months**  IDA= Hb <11g/dL and 2 abnormal iron markers (MCV<74 fl, RDW>14% , ZPP/H>69 µmol/mol, EP> 80 µg/dL, TSAT < 12% and ferritin <12 µg/L  ID= Hb >= 11.5g/dL and 2 abnormal iron markers  IS= Hb >=11.5g/dL and no more than 1 abnormal iron marker. | 2008  IDA (28), ID (28), IS (21)  2009  IDA (4)  IS (5) | **Motor**  2008  BSID PDI, PDMS-2, INFANIB, Bimanual co-ordination task.    2009  Reach and Grasp task | **Motor**  2008  When adjusted for confounders, ID and IDA groups scored worse on some assessments but not all.  2009  IDA infants had less straight, slower movements than IS group. | **High**  Small sample size, multiple comparisons, inadequate adjustment for confounders. |
| Tofail et al.  (2013) [38] | 434 | Bangladesh (LMIC) | **6-24 months of age**  IDA= sTfR ≥5.0 mg/L and Hb >8 and <11g/dL  IS= sTfR <5.0 mg/L and Hb>=11g/dL | IDA (225)  IS (209) | **Cognitive**  BSID MDI  **Motor**  BSID PDI | **Cognitive & Motor**  When adjusted for confounders, no difference in scores between IDA and IS groups. | **Low**  Large sample, adjusted for relevant confounders, blinded to iron group.  However, no other micronutrients measured. |
| **2-4 years** | | | | | | | |
| Gashu et al. (2016) [35] | 541 | Ethiopia (LMIC) | **54-60 months**  IDA= ferritin <12 µg/L or ferritin<30 µg/L if AGP>1.2g/L  & Hb <11g/dL  ID= ferritin <12 µg/L or <30 µg/L if AGP>1.2g/L  IS= ferritin >=12 µg/L or >=30 µg/L if AGP 1.2g/L & Hb>=11g/dL | ID (49)  IDA (29)  IS (463) | **Cognitive**  WPPSI-III and school readiness test | **Cognitive**  No difference in scores between IDA, ID or IS groups, | **Medium**  Reasonable sample size, multiple regression, assessment of some nutritional markers. |
| LMIC- low and middle-income country, HIC- high income country, ID- Iron deficiency, IDA- Iron deficiency anaemia , IS- Iron sufficient, Hb- Haemoglobin, MRI- Magnetic resonance imaging, BERA- Brain Stem Evoked Response Audiometry, ABR- auditory brainstem response, VEP- visually evoked potential , BSID- Bayley Scales of Infant and Toddler Development, MDI- Mental Development Index, PDI- Psychomotor Development Index, IBR- Infant Behaviour Rating, BRS- Behaviour rating Scale , PDMS- Peabody Developmental Motor Scale, INFANIB- Infant Neurological International Battery, WPSII-III- Wechsler Pre-school and Primary Scale of Intelligence , MSEL- Mullen Scales of Early Learning, ELC- Early Learning Composite, TBAQ-R- Toddler Behaviour Assessment Questionnaire-R, MCV- Mean corpuscular volume, RDW- Red cell distribution width, MCH- Mean corpuscular haemoglobin, MCHC- Mean corpuscular haemoglobin concentration, TSAT- Transferrin Saturation, AGP- alpha-1 acid glycoprotein, ZPP/H- Zinc protoporphyrin/Haem, CRP- C- reactive protein, TIBC- Total iron binding capacity , FEP- Free erythrocyte protoporphyrin, IBC- Iron binding capacity. | | | | | | | |

| **Table S4 Longitudinal Studies** | | | | | | | |
| --- | --- | --- | --- | --- | --- | --- | --- |
| **Author (Year)** | **Total Sample Size** | **Setting** | **Iron Measures taken (Timing)** | **Comparison groups (n) at outcome** | **Outcome Measure (Timing)** | **Results** | **Risk of Bias** |
| **Pregnancy/ Delivery** | | | | | | | |
| ElAlfy et al.**(2018) [25] | 50 | Egypt (LMIC) | **Cord blood at delivery**  ID= ferritin 11-75 µg/L  IS= ferritin >=75 µg/L | ID (36)  IS (14) | **Physiological**  ABR Screening Test  (New-born)  ABR Diagnostic Test  (Aged 3 months) | **Physiological**  New-born  ID infants were more likely to fail the ABR test.  Aged 3 months  All interpeak latencies were longer in ID infants compared to IS infants. | **High**  Small sample, no adjustment for confounders, multiple comparisons, unknown relevance of difference in outcome measure. |
| Geng et al. (2015) [26]  Armony-Sivan et al.*  (2016) [57] | 254 | China (LMIC) | **Cord blood at delivery**  ID = ZPP/H >118 μmol/mol  IS= ZPP/H <=118 μmol/mol | ID (35)  IS (92) | **Cognitive**  EEG recognition memory assessment  (Aged 2 months) | **Cognitive**  IS responses indicated recognition memory, ID did not show recognition memory. | **Medium**  Low sample size,  Some adjustment for confounders, high loss to follow up |
| Lou et al.  (2016) [24] | 115 | China (LMIC) | **Maternal blood in 3^rd^ trimester**  Maternal ID= ferritin <16 µg/L  Maternal IS= ferritin >=16µg/L  **Cord blood at delivery**  Infant ID= ferritin <75 µg/L  Infant IS >=75 µg/L | Maternal ID (49)  Maternal IS (19)  Infant ID (31)  Infant IS (38) | **Physiological**  ABR (48)  (Aged 3 months)  ABR (45)  (Aged 10 months) | **Physiological**  3 months  No difference between maternal ID and maternal IS groups or infant ID and infant IS groups.  10 months  No difference between maternal ID and maternal IS groups, infant ID group showed differences in some wave latencies but not others. | **Medium**  Small sample size,  basic adjustments included corrected for multiple comparisons. |
| Mireku et al (2016) [28] | 828 | Benin (LMIC) | **Maternal blood up to 28 weeks gestation (AN1) and 1 month later (AN2)**  ID= ferritin <12 µg/L or < 70 µg/L in the presence of inflammation.  IS = ferritin>12 µg/L or >70 µg/L in the presence of inflammation | AN1  ID (191)  IS (475)  AN2  ID (213)  IS (414) | **Cognitive**  MSEL ELC  **Motor**  MSEL Gross Motor Scale  (Aged 12 months) | **Cognitive and Motor**  No difference in scores based on antenatal iron status. | **Low**  Large sample, adjusted for confounders, well recognised assessment, adapted for setting, post hoc powered to detect small difference outcome. |
| Tran et al. (2014) [27] | 523 | Vietnam (LMIC) | **Maternal blood in early and late pregnancy**  ID= ferritin < 15 µg/L or < 30 µg/L if CRP>5 mg/L  IS= ferritin >=15 µg/L or >=30 µg/L if CRP>5 mg/L | Early pregnancy  ID (17)  IS (411)  Late pregnancy  ID (182)  IS (236) | **Motor**  BSID PDI  (Aged 6 months) | **Motor**  No direct relationship between motor scores and ID, however there as an indirect relationship via anaemia in late pregnancy. | **Low**  Large sample size, adjusted for relevant confounders (excluding other micronutrients), sophisticated analysis, but few ID in early pregnancy. |
| **0-6months** | | | | | | | |
| No studies found | | | | | | | |
| **6-24 months** | | | | | | | |
| Beltran-Navarro et al* (2012) [34] | 58 | Mexico (LMIC) | **6 and 14-18 months of age**  IDA= Hb <11g/dl and ferritin <12 µg/L  ID= Hb >=11 g/dL and ferritin <12 µg/L  IS= Hb >=11g/dl and ferritin >=12 µg/L | IDA (9)  ID (17)  IS (30) | **Cognitive**  BSID MDI  Auditory verbal/nonverbal perception  Preschool Language Scales  (Aged 14-18months) | **Cognitive**  No differences between IDA, ID or IS groups in any assessments | **High**  Small sample size, no adjustment for confounding factors |
| Burden et al.  (2007) [94]  Lozoff et al. (2008) [95]  Carter et al. (2010) [39] | 242 | USA (HIC)  Assessed at 9 months, given iron therapy then assessed at 12 months | **9 and 12 months of age**  IDA= Hb<10.5g/dL and 2 or more abnormal iron markers (MCV<74fL, RDW >14%, ZPP/ Heme >69µmol/mol, ferritin <12µg/L, TSAT<12%)  IS= Hb ≥11.5 g/dL and no more than 1 abnormal iron marker | 2007  9 months of age  IDA (13)  IS (15)  12months of age  IDA (9)  IS (11)  2010  9 months of age  IDA (28)  IS (21) | 2007  **Cognitive**  EEG recognition memory assessment  2010  **Cognitive**  Fagan test for infant intelligence, A not B task, object permanence,  **Socio-emotional**  Emotionality, Activity, and Sociability Temperament Survey, examiner reported behaviour rating scale.  (Aged 9 and 12 months) | 2007  **Cognitive**  At 9 and 12 months of age,  IS group showed expected responses, IDA group did not.  2010  **Cognitive**  Inconsistent findings at 9 months of age, IDA scored worse on some but not all tasks.  No difference in scores between groups at 12 months of age.  **Socio-emotional**  IDA scored worse than IS on some scales, no difference on others at both 9 and 12 months. | **High**  Very small sample size, multiple comparisons, inadequate adjustment for confounders, difficult to interpret results. |
| Doom et al. (2014) [33] | 55 | USA (HIC)  National and international Adoptees | **18-36 months of age (soon after adoption)**  IDA= Hb< 11g/dL and 2 or more abnormal iron markers (TSAT < 12%, MCV<74 fL, ferritin<12 µg/L, and IBC > 538 µg/dL)  ID= Hb>=11g/dL, and 1 or more abnormal iron markers  IS= Hb>=11g/dL and no abnormal iron markers. | IDA (11)  ID (16)  IS (28) | **Cognitive**  MSEL, Delayed Gratification Task, Dimensional Change Card Sort, Spin the Pots  (11-14 months after baseline) | **Cognitive:**  Both ID and IDA groups scored consistently worse than IS group. | **High**  Very small sample size, little adjustment for other factors, lots of unknowns about infant history, no follow up on iron status, wide range of ages, no home environment factors. |
| Fuglestad et al (2013) [41] | 57 | USA (HIC) | **8-46 months of age and 6months later**  ID= 2 or more abnormal indices: TSAT<12 %, ferritin <12 µg/L, and MCV< 74 fL  IDA= 2 or more abnormal indices and Hb<11g/dL | Baseline  IS (42)  ID (15)  End line  IS (47)  ID (10) | **Cognitive**  MSEL ELC  **Socio-emotional**  TBAQ-R | **Cognitive**  Lower ELC score in ID group at follow up only, mediated by hyperactivity.  **Socio-emotional**  No overall difference between groups, ID at end line were more hyperactive. | **High**  Small sample size, little adjustment for confounders, very wide age range. |
| Fuglestad et al. (2016) [40] | 58 | USA (HIC)  National and International Adoptees | **8-18 months of age and 6 months later**  IDA= Hb>=11g/dL, and 2 or more abnormal iron markers (TSAT< 12%, MCV<74 fL, ferritin<12 µg/L, and IBC > 538 µg/dL)  IS= Hb>=11g/dL and no abnormal iron markers. | Baseline IDA (8)  Baseline IS (50)  End line IDA (3)  End line IS (55) | **Cognitive**  BSID, elicited imitation task  **Physiological**  VEP  (Aged 14-24 months) | **Cognitive and Physiological**  ID group had lower BSID scores and longer latencies on VEP compared to IS group. | **High**  Very small sample size, infants from disparate backgrounds, little adjustment for confounding factors. |
| Walter et al.  (1989, 1989) [32,53] | 196 | Chile (LMIC) | **Mean of aged 9, 12- and 15-months samples**  IDA= Hb<11g/dL and 2 or more abnormal iron markers (MCV<70fL, FEP >=100 µg/dL red blood cells, ferritin <12µg/L)  ID= Hb >=11g/dL, 2 or more abnormal iron markers | IDA (39)  ID (127)  IS (30)  (mean values from 9, 12 and 15 months) | **Cognitive**  BSID MDI  (12 months) | **Cognitive**  IDA group scored lower than ID and IS groups.  **Motor**  IDA group scored lower than ID. ID scored lower than IS. | **Medium**  Reasonable sample size but no adjustment for confounding factors. |
| **Duration Comparison** | | | | | | | |
| **Author (Year)** | **Total Sample Size** | **Setting** | **Iron measurements (Timing)** | **Comparison groups (n) in outcome analysis** | **Outcome Measure (Timing)** | **Results** | **Risk of Bias** |
| Armony-Sivan et al. (2016) * [57] | 80 | China (LMIC) | **Cord blood at delivery and aged 9 months**  Prenatal ID= ferritin<75 μg/L or ZPP/H >118 μmol/mol  Postnatal ID= MCV <74 fl, RDW >14.5%, ferritin <12.0, ZPP/H >69 μmol/mol haem | Prenatal ID only (21)  Postnatal ID only (20)  Pre and postnatal ID (9)  No ID (30) | **Socio-emotional**  EEG response to positive, negative and neutral stimuli. | **Socio-emotional** No effect of iron group in response to positive, negative or neutral stimuli. ‘Pre and postnatal ID’ showed different pattern of response compared to other groups. | **Medium**  Confounding factors well thought through, study powered don’t know full relevance of the assessment, very small sample size in ‘pre- and post-natal’ group. |
| Beltran-Navarro et al. (2012) *[34] | 58 | Mexico (LMIC) | **6 and 14-18months**  ID = ferritin <12 µg/L  IS= ferritin >=12 µg/L  Chronic ID= ID or IDA at 6 and 14-18months  Never ID= IS at 6 and 14-18months | Chronic ID (6)  Never ID (24) | **Cognitive**  BSID, Auditory verbal/ non-verbal task.  Preschool Language Scales  (Aged 14-18 months) | **Cognitive**  No difference between Chronic ID and never ID groups on cognitive measures.  Chronic ID group scored lower on preschool language scale and BSID language scales  **Motor**  Chronic ID scored lower in BSID PDI | **High**  Very small sample size, no adjustment for other factors. |
| Santos et al. (2018) [52] | 1194 | China (LMIC) | **Cord blood at delivery and aged 9 months**  Prenatal ID= ferritin < 75 µg/L or ZPP/H > 118 µmol/mol  Postnatal ID= body iron < 0 mg/kg, calculated using ferritin and sTfR | Prenatal ID (256)  Postnatal ID (288)  Pre- and post-natal ID (253)  No ID (397) | **Motor**  PDMS-2, BSID motor quality scale  (Aged 9 months) | **Motor**  Prenatal only and postnatal only ID scored worse than no ID group on gross motor tasks. ID in both periods didn’t further impact scores. | **Low**  Large sample size, good range of confounders considered, excluding other micronutrients. |
| *Included in specific exposure window sections as well as duration comparison. **Main results of paper compare anaemic and non-anaemic mothers and not included in the review. LMIC- low and middle- income country, HIC- high income country, ID- Iron deficiency, IDA- Iron deficiency anaemia , IS- Iron sufficient, Hb- Haemoglobin, MRI- Magnetic resonance imaging , BERA- Brain Stem Evoked Response Audiometry, ABR- auditory brainstem response, VEP- visually evoked potential , BSID- Bayley Scales of Infant and Toddler Development, MDI- Mental Development Index, PDI- Psychomotor Development Index, IBR- Infant Behaviour Rating, BRS- Behaviour rating Scale , PDMS- Peabody Developmental Motor Scale, INFANIB- Infant Neurological International Battery, WPSII-III- Wechsler Pre-school and Primary Scale of Intelligence , MSEL- Mullen Scales of Early Learning, ELC- Early Learning Composite, TBAQ-R- Toddler Behaviour Assessment Questionnaire-R, MCV- Mean corpuscular volume, RDW- Red cell distribution width, MCH- Mean corpuscular haemoglobin, MCHC- Mean corpuscular haemoglobin concentration, TSAT- Transferrin Saturation, AGP- alpha-1 acid glycoprotein, ZPP/H- Zinc protoporphyrin/Haem, CRP- C- reactive protein, TIBC- Total iron binding capacity , FEP- Free erythrocyte protoporphyrin, IBC- Iron binding capacity. | | | | | | | |

| **Table S5 Intervention Studies** | | | | | | | | | | | | | | |
| --- | --- | --- | --- | --- | --- | --- | --- | --- | --- | --- | --- | --- | --- | --- |
| **Author (Year)** | **Full Sample Size** | | **Setting** | **Study Design (n) In outcome groups** | | **Baseline Cognitive Differences** | | **Intervention Comparison Groups** | | **Supplementation Efficacy** | | **Outcome Measure (Timing)** | **Results** | |
| **Supplementation in Pregnancy** | | | | | | | | | | | | | | |
| Angulo- Barroso et al.*  (2016) [54] | 1276 dyads | China (LMIC) | | | 4 arm RCT:  Antenatal iron (316), infant iron (321), antenatal and infant iron (327), no iron (312) | | NA | | **Antenatal Iron**  Iron (30mg) + Folic acid (400µg/d)  **Control**  Folic acid (400µg/d)  Daily from <20 weeks GA to delivery | No impact on infant iron status | **Motor**  PDMS,  BRS Motor Quality Factor.  (Aged 9 months) | | | **Motor**  No difference in scores between antenatal iron group and control group. |
| Zhou et al.  (2006) [51] | 430 dyads | Australia  (HIC) | | | 2 arm RCT:  Iron (153) Placebo (149) | | NA | | **Antenatal Iron** 20mg Iron  **Control**  Placebo tablet  Daily from 20 weeks GA to delivery | Supplementation significantly improved maternal iron status. Infant iron status not reported. | **Cognitive**  Stanford Binet Intelligence Scale  **Socio-motional**  Strengths and Difficulties questionnaire  (Aged 4 years) | | | **Cognitive**  No difference in scores between antenatal iron and control groups  **Socio-emotional** No difference in mean scores between groups on any scale or total, but higher % of iron group scored in ‘abnormal’ range. |
| **Supplementation from birth to 6 months** | | | | | | | | | | | | | | |
| Angulo-Barroso et al.*  (2016) [54] | 1276 dyads | China (LMIC) | | | 4 Arm RCT:  Antenatal iron (316)  Infant iron (321) Antenatal + infant iron (327) No iron (312) | | NA | | **Iron**  1mg/kg oral iron solution  **Control**  Placebo oral solution  Daily from 6 weeks of age for 7.5 months | Not reported | **Motor**  PDMS,  BRS, Motor Quality Factor.  (Aged 9 months) | | | **Motor**  Infant iron group had better motor skills at 9 months compared to no iron group and iron in pregnancy group. |
| Friel et al.  (2003) [60] | 77 | Canada (HIC) | | | 2 arm RCT:  Iron (26)  Placebo (20) | | NA | | **Iron**  7.5mg iron syrup daily  **Control**  Placebo syrup  Daily from 1month of age for 5 months | Supplementation reduced prevalence of IDA at 6 months (7% vs 33%) | **Cognitive**  BSID MDI  Teller Visual Acuity Cards  **Motor**  BSID PDI  (Aged 12-18 months) | | | **Cognitive**  No difference in scores between groups.  **Motor**  Iron group had better motor skills at 12-18 months of age. |
| Moffatt et al.  (1994) [55] | 283 | Canada (HIC) | | | 2 arm RCT:  Iron-enriched Formula (n not stated)  Control Formula (n not stated) | | NA | | **Iron**  Iron enriched formula (12.8mg/L)  **Control**  Regular formula (1.1mg/L)  Ad libitum  from 2 months. no end point reported. | All measures of iron significantly improved in iron-enriched group by 6 months. Chronic iron depletion lower in supplemented group (26.4% vs 52.8%). | **Cognitive**  BSID MDI  **Motor**  BSID PDI  **Socio-Emotional**  BSID IBR  (Aged 6, 9, 12 and 18months) | | | **Cognitive**  No difference in groups from 6-18months  **Motor**  Iron group had better motor scores at 9 and 12 months. No difference at 18months.  **Socio-emotional**  No difference between groups from 6-18 months |
| Otero et al.  (2019) [61] | 50 | Mexico  (LMIC) | | | 1.Comparison of scores in IDA (25) and non-IDA (25) before treatment.  2. Comparison of IDA infant scores before (25) and after (25) supplementation | | IDA infants had abnormal EEG responses at baseline, compared to non-IDA controls | | **Iron**  5mg/kg iron  **No control group**  Daily  from 3-12 months of age for 4 months | Mean Hb increased from 8.2 to 12.4g/dL- mostly iron replete by end line. | **Physiological**  Quantitative EEG activity.  (Aged 7-16 months) | | | **Physiological**  EEG responses normalised following supplementation. |
| **Supplementation from 6-24months** | | | | | | | | | | | | | | |
| Akman et al. (2004) [66] | 108 | Turkey (LMIC) | | | Before and after supplementation  IDA (37)  ID (21 iron/ 19 no iron)  IS control at baseline (31) | | Lower mental and motor scores in ID and IDA at baseline compared to IS group. | | **Iron**  6mg/kg iron  (All IDA + half of ID group)  **Control**  ID control: no supplement  No IDA control (All supplemented)  Daily from 6-30 months of age for 3months | IDA-mean Hb increased with supplementation  ID- Hb increased more in iron group compared to control group. | **Cognitive**  BSID MDI,  Denver developmental screening test  **Motor**  BSID PDI  (Aged 9-33 months) | | | **Cognitive**  Supplemented groups scored on par with IS group at end line  **Motor**  Supplemented group scored on par with IS at end line. |
| Aukett et al.  (1986) [69] | 110 | UK  (HIC) | | | 2 arm RCT in anaemic infants:  Iron (54)  Placebo (56) | |  | | **Iron**  24mg iron+ 10mg Vitamin C  **Control**  10mg Vitamin C  Daily from 17 months of age for 2 months | Only 6 supplemented participants still IDA at end line | **Motor**  Subset of DDST  (Aged 19 months) | | | **Motor**  No difference in motor scores between groups. More infants in iron group reached ‘expected developmental level’ at endline. |
| Black et al.  (2004) [68] | 346 | Bangladesh (LMIC) | | | 5 arm RCT:  Iron (49)  Zinc (49),  Iron+ Zinc (43)  MMN (35)  Riboflavin (45) | |  | | **Iron**  20mg iron +1mgRiboflavin    **Control**  1mg Riboflavin  Weekly from 6 months of age for 6 months. | No difference in end line Hb between iron and control group. | **Cognitive**  BSID MDI  **Motor**  BSID PDI  **Socio-emotional**  BSID BRS  (Aged 12 months) | | | **Cognitive**  No difference between groups.  **Motor**  No difference between groups.  **Socio-emotional**  No difference between groups |
| Idjradinata et al.  (1993) [65] | 126 | Indonesia (LMIC) | | | 2 arm RCT in IDA, ID and IS infants:  Iron  IDA (25), ID (14), IS (24)  Placebo  IDA (25), ID (15), IS (23) | | IDA participants had lower mental and motor scores than ID and IS at baseline, no difference between ID and IS. | | **Iron**  3mg/kg iron  **Control**  Placebo  Daily from 12-18months of age for 4 months | Supplementation increased Hb of IDA/ ID participants to least 11g/dL | **Cognitive**  BSID MDI  **Motor**  BSID PDI  (Aged 16-22months) | | | **Cognitive**  Supplementation increased scores among IDA group only.  **Motor**  Supplementation increased scores among IDA group only. |
| Iglesias-Vazquez et al.  (2019) [71] | 142 | Spain (HIC) | | | 2 arm RCT 1:3  Iron-enriched Formula (102) Regular Formula (28) | |  | | **Iron**  Iron-enriched formula (1.2mg/100ml)  **Control**  Regular formula (0.4mg/100ml)  Ad libitum from 6 months of age for 6 months | Fewer cases of iron depletion, ID and IDA in iron-enriched group at 12 months. No difference in serum iron. | **Cognitive**  BSID MDI  **Motor**  BSID PDI  (Aged 12 months) | | | **Cognitive**  No difference between groups at 12 months of age.  **Motor**  No difference between groups at 12 months of age. |
| Kurekci et al.  (2006) [47] | 132 | Turkey (LMIC) | | | Before and after supplementation study in ID (24) and IDA (25) participants.  IS controls at baseline (44) | | No difference between ID, IDA or IS controls at baseline | | **Iron**  ID and IDA groups received 5mg/kg iron  **No control group**  Daily from 6-24 months of age for 3months | Iron parameters equivalent to IS controls at end line. | **Physiological**  Brainstem auditory evoked potentials  (Aged 9-27months) | | | **Physiological**  No difference between scores before and after supplementation. |
| Lind et al. (2004) [56] | 680 | Indonesia (LMIC) | | | 4 arm RCT:  Iron +Vitamin C (166)  Zinc + Vitamin C (167)  Iron + Zinc + Vitamin C (164)  Vitamin C (169) | |  | | **Iron**  10mg iron + 30mg Vitamin C  **Control**  Vitamin C  Daily from 6 months of age for 6 months | Higher mean Hb and ferritin, lower sTfR and lower prevalence of anaemia in iron group at end line. | **Cognitive**  BSID MDI  **Motor**  BSID PDI  **Socio-emotional**  BSID BRS  (Aged 12months) | | | **Cognitive**  No difference between groups.  **Motor**  Higher scores in iron group compared to control group than control at 12 months.  **Socio-emotional**  No difference in groups. |
| Lozoff et al.  (1982, 1982, 1985) [63, 91, 92] | 69 | Guatemala  (LMIC) | | | 2 arm RCT in IDA and non-IDA groups.  Iron  IDA (15), non-IDA (19)  Placebo  IDA (13), non-IDA (21) | | Lower cognitive and motor scores in IDA group compared to non- IDA group at baseline. | | **Iron**  5mg/kg iron  **Control**  Placebo  Twice daily from 6-24 months of age for 7-10 days | Increased Hb in supplemented IDA group, no change in any other group. | **Cognitive**  BSID MDI  **Motor**  BSID PDI  (1 week later) | | | **Cognitive**  No difference between supplementation groups.  **Motor**  No difference between supplementation groups. |
| Lozoff et al.  (1987) [64] | 191 | Costa Rica  (LMIC) | | | 3 arm RCT in 52 IDA participants:  IV iron/  Oral iron/ Placebo  2 arm RCT in 132 non-IDA participants:    Oral iron /Placebo | | Moderately IDA (Hb<10g/dL) scored worse than mild or non-IDA in cognitive tests. Mildly IDA (Hb<10.5g/dL) scored worse than non-IDA in motor tests. | | **Iron**  1-week 10mg/kg iron +12 weeks 6mg/kg daily  Or  IV iron dosed for iron repletion at 12-23 months (IDA only)    **Control**  Placebo  From 12-23 months of age for 3months | 36% of supplemented IDA were IS by the end line. Those who didn’t become IS started with worse iron status. | **Cognitive**  BSID PDI  **Motor**  BSID PDI  (Aged 15-26 months) | | | **Cognitive**  Increased scores in moderate IDA participants who became IS after 3 months, but no difference between full groups  **Motor**  Increased scores in IDA participants who became IS after 3 months. No difference between full groups. |
| Lozoff et al. (1996) [72] | 86 | Costa Rica (LMIC) | | | 1) Before and after supplementation study in IDA participants (32).  2) RCT in non-IDA participants:  Iron (27)  Placebo (27) | | IDA group scored lower than non-IDA on cognitive test. Motor scores were equivalent between groups. More IDA infants were more wary and fearful, but no difference overall. | | **Iron**  6mg/kg iron  **Control**  Placebo (non-IDA only)  Daily from 12-23 months of age for 6 months | No difference in iron status between IDA and non-IDA by end line, but IDA group still had poorer iron status than non-IDA at midline. | **Cognitive**  BSID MDI  **Motor**  BSID PDI  **Socio-emotional**  BSID IBR  (Aged 15-26 months and aged 18-29 months) | | | **Cognitive**  IDA scored lower than non-IDA at midline, but only trend level difference at end line.  No difference in scores between non-IDA groups.  **Motor:**  No difference in scores between IDA or non-IDA supplementation groups  **Socio-emotional:**  No overall difference, heightened wariness and fearfulness among IDA at baseline no longer significant at end line. |
| Lozoff et al. (2003) [50] | 1657 | Chile (LMIC) | | | 2 arm RCT in non-anaemic participants:  Iron-enriched formula (1123)  Regular formula (534) | |  | | **Iron**  High-iron formula (12.7mg/l)  Or  Low-iron formula (2.3mg/l)  **Control**  Iron free formula  Ad libitum from 6 months of age for 6 months. | IDA lower in high iron group at end line (3.1% vs 22.6%)  No difference between high vs low iron groups after | **Cognitive**  BSID MDI,  Fagan Test of Infant Intelligence  **Motor**  BSID PDI  Motor Milestones  **Socio-emotional**  BSID BRS  (Aged 12 months) | | | **Cognitive**  No difference between groups on total score but control group had longer looking time.  **Motor**  No difference between total score but control group learnt to crawl later than iron group.  **Socio-emotional**  Control group scored worse on some scales, no difference in others. |
| Lozoff et al.  (2010) [48] | 61 | USA (HIC) | | | Before and after supplementation study in IDA (19) non-IDA (42). | | IDA had fewer eye blinks per minute than non-IDA group. | | **Iron**  22mg iron  **No control group**  Daily from 9-10 months of age for 3 months | Hb increased in IDA group at end line compared to baseline. | **Physiological**  Eye blink rate assessment  (Aged 12 months) | | | **Physiological**  Eye blink rate increased after supplementation in IDA group. By end line no difference between IDA and non-IDA groups. |
| Morley et al.  (1999) [70] | 493 | UK (HIC) | | | 3 arm RCT:  Iron-enriched formula (90)  Regular formula (76)  Cow’s milk (95) | |  | | **Iron**  Iron-enriched formula (1.2mg/L iron)    **Control**  Regular formula (0.9mg/L iron)  Ad libitum from 9 months of age for 9 months | No difference in prevalence of anaemia or sTfR concentration between groups. Ferritin higher in iron-enriched group. | **Cognitive**  BSID MDI  **Motor**  BSID PDI  (Aged 18 months) | | | **Cognitive**  No difference between groups.  **Motor**  No difference between groups. |
| Oski et al. (1978) [67]  Honig et al. (1978) [77] | 24 | USA (HIC) | | | 2 arm RCT in IDA participants:  Iron (12)  Placebo (12) | | No difference in scores between groups at baseline. | | **Iron**  iron injection calculated to replenish stores    **Control**  placebo injection  Single dose at 9-26 months of age | No iron measurements after supplementation | **Cognitive**  BSID MDI  **Motor**  BSID PDI  **Socio-emotional**  BSID IBR  (1 week later) | | | **Cognitive**  Greater increase in score in supplemented group.  **Motor**  No difference in motor score between groups.  **Socio-Emotional**  No Report of socio-emotional scale. |
| Oski et al.  (1983) [76] | 38 | USA (HIC) | | | Before and after supplementation study in Iron depleted (10), ID (18) and IS (10) groups. | | No difference in baseline scores between groups | | **Iron**  50mg iron  **No control group**  Single dose at 9-12 months of age | Iron depletion corrected by supplementation | **Cognitive**  BSID MDI  **Motor**  BSID PDI  **Socio-emotional**  BSID IBR  (1 week later) | | | **Cognitive**  Greater increase in scores in iron deficient group than others.  **Motor**  No report of motor scale  **Socio-Emotional**  Not report of socio-emotional scale |
| Sarici et al.  (2001) [46] | 40 | Turkey (LMIC) | | | 1) Baseline comparison of IDA (20) and IS (20)  2) Before and after supplementation study in IDA only (20) | | No difference in scores between IS and IDA at baseline | | **Iron**  IDA group 4mg/kg iron daily  **No control group**  Daily from 6-24months of age for 3 months | Improved Hb, haematocrit, and MCV following supplementation. | **Physiological**  Brainstem auditory evoked potentials  (Aged 9-27months) | | | **Physiological**  No difference in scores following supplementation. |
| Walter et al. (1983) [75] | 51 | Chile (LMIC) | | | Before and after 10 days iron supplementation in IDA, ID and IS infants  IDA (11)  ID (15)  IS (12) | | Lower MDI in IDA group, no other differences. | | **Iron**  3-4mg/kg iron  **No control group**  Daily from 15 months of age for 14 days. | Not reported | **Cognitive**  BSID MDI  **Motor**  BSID PDI  **Socio-emotional**  BSID IBR | | | **Cognitive**  Improved scores following supplementation in IDA group only.  **Motor**  No impact of supplementation on scores.  **Socio-emotional**  Among IDA group, there was an improvement in scores on some scales following supplementation. No improvement in ID or IS infants. |
| Walter et al. 1989** [32] | 196 | Chile (LMIC) | | | Before and after supplementation study Compared BSID scores before and after 10 days of Iron supplementation (196) | | Lower mental and motor scores in IDA compared to ID and IS groups. | | **Iron**  10 days iron supplementation    **Control**  Placebo  (IDA only)  Followed by daily supplementation  from 12 months of age for 3 months-  dose not specified. | No record of impact of 10-day iron supplementation  3 months iron supplementation reversed anaemia in all infants but only corrected ID in 11/39 anaemic infants. | **Cognitive**  BSID MDI  **Motor**  BSID PDI  Midline (+ 10 days)  End line (+3 months) | | | **Cognitive**  No difference between iron and placebo group following 10 days or 3 months of supplementation.  **Motor**  No difference between iron and placebo group following 10 days or 3 months of supplementation**.** |
| **Supplementation from 2-4 years** | | | | | | | | | | | | | | |
| Kabakus et al.  (2002) [49] | 30 | Not Reported | | | Before and after supplementation study in IDA participants (18)  Non-IDA controls at baseline (12) | | Slower nerve conduction in IDA compared to non-IDA group at baseline | | **Iron**  6mg/kg daily  from 25-42months of age for 3months  **Control**  Non-supplemented non-IDA at baseline. | Iron parameters in IDA group comparable to non-IDA controls after supplementation | **Physiological**  Nerve conduction velocity  (Aged 28-45 months) | | | **Physiological**  At end line, supplemented group performed comparably to non-IDA group’s baseline scores. |
| Metallinos-Kataras et al.  (2004) [78] | 49 | Greece (HIC) | | | 2 arm cluster RCT in IDA and non-IDA participants:  Iron  IDA (14), non-IDA (18)  Placebo  IDA (7), non-IDA (10) | | IDA performed learning tasks better than non-IDA at baseline, no other differences | | **Iron**  15mg iron + MMN  **Control**  non-iron containing MMN  Daily from 36-59 months of age for 3 months | Increase in Hb in iron group but no difference in ferritin or TSAT compared to control group. | Cognitive  Battery of computer- based assessments  (Aged 39-62 months) | | | **Cognitive**  Improved reaction time and processing speed among supplemented IDA group, but no difference in other tasks.  No impact of supplementation in non-IDA group. |
| *Trial included antenatal and infant supplementation arms and is included in both relevant sections. **Paper also included in longitudinal section. RCT- Randomised controlled trial, PDMS-2- Peabody Developmental Motor Scale, ID- Iron deficiency, IDA- Iron deficiency anaemia, IS- Iron sufficient ,GA- Gestational age, BSID- Bayley Scales of Infant and Toddler Development, MDI- Mental development index, PDI- Psychomotor Development Index, IBR- Infant behaviour record , BRS- Behaviour rating scale, EEG- electroencephalography, DDST- Denver developmental screening test, LMIC- low and middle- income country, HIC- high income country, Hb- Haemoglobin, TSAT- Transferrin saturation, MMN- Multiple micronutrient, sTfR- Soluble transferrin receptor, MCV- mean corpuscular volume. | | | | | | | | | | | | | | |

| **Table S6 Risk of Bias** | | | | | | | | | | | |
| --- | --- | --- | --- | --- | --- | --- | --- | --- | --- | --- | --- |
| **Author (Year)** | **Iron Deficiency Prevalent?** | **Utility of Control Group** | **Utility of Intervention** | **Utility of outcome** | **Randomised/ Groups differences** | **Blinded?** | **Loss to follow up** | **Treatment effect (iron)** | **Treatment effect (brain)** | **Sample Size** | **Overall Risk of Bias.** |
| **Supplementation in pregnancy** | | | | | | | | | | | |
| Angulo-Barroso (2016) [54] | Yes | Good | Self-administered | Good | Yes, successful | Double | Small | No impact on infant | No effect | Large- powered | Low |
| Zhou  (2006) [51] | Yes | Good | Good | Good | Yes, successful | Double | Medium- no differences between lost/ retained | Impact on infant not reported | No effect | Large-powered | Low |
| **Supplementation from 0- 6 months** | | | | | | | | | | | |
| **Author (Year)** | **Iron Deficiency Prevalent?** | **Utility of Control Group** | **Utility of Intervention** | **Utility of outcome** | **Randomised/ Groups differences** | **Blinded?** | **Loss to follow up** | **Treatment effect (iron)** | **Treatment effect (brain)** | **Sample Size** | **Overall Risk of Bias.** |
| Angulo-Barroso (2016) [54] | Yes | Good | Good | Good | Yes, successful | Double | Small | Impact | Small- medium | Large powered | Low |
| Friel (2003) [60] | Unclear | Good | Good | Good | Yes, successful | Double | Large | Impact | Large-motor only | Small – not powered | Medium |
| Moffatt (1994) [55] | Yes | Formula- low dose iron | Formula  non-WHO compliant | Good | Yes, successful | Double | Medium | Impact | Motor- 9-12months only | Large- powered | Low |
| Otero (2019) [61] | Yes | Poor | Fair – wide age range | Fair | No- unadjusted for differences | No | Small | Impact | Large | Small- not powered | High |
| **Supplementation from 6-24 months** | | | | | | | | | | | |
| **Author (Year)** | **Iron Deficiency Prevalent?** | **Utility of Control Group** | **Utility of Intervention** | **Utility of outcome** | **Randomised/ Groups differences** | **Blinded?** | **Loss to follow up** | **Treatment effect (iron)** | **Treatment effect (brain)** | **Sample Size** | **Overall Risk of Bias.** |
| Akman (2004) [66] | Yes | Poor- no placebo | Fair- wide age range | Good | Yes | Single | Small | Impact | Small | Small- not powered | High |
| Aukett (1986) [69] | Yes | Good | Good | Good | Yes | Double | Small | Impact | No effect | Medium- not powered | Medium |
| Black  (2004) [68] | Yes | Good | Good | Good | Yes | Double | Large | No impact | No effect | Large- powered for large effect | Medium |
| Idjradinata (1993) [65] | Yes | Good | Good | Good | Yes | Not reported | Not reported | Impact | Large | Medium- not powered | Medium |
| Iglesias- Vazquez (2019) [71] | No | Formula- low dose iron | Formula- ad libitum | Good | Yes | Double | Small | Impact | No effect | Medium- not powered | Medium |
| Kucekci (2006) [47] | Not reported. | Poor | Fair– wide age range | Fair | No | No | Small | Impact | No effect | Small- not powered | High |
| Lind  (2004) [56] | Yes | Good | Good | Good | Yes | Double | Small | Impact | Small | Large- powered for medium effect | Low |
| Lozoff  (1982, 1982,1985)  [63,91,92] | Yes | Good | Poor- wide age range and short course | Poor- 1 week after baseline | Yes | Double | Small | Impact | No effect | Medium- powered to detect large difference | Medium |
| Lozoff (1987) [64] | Yes | Good | Very good- observed | Good | Yes | Double | Small | Some impact | Sub-group effect | Medium- not powered | Medium |
| Lozoff (1996) [72] | Yes | Fair- IDA not randomised, other were | Very good- observed | Good | IDA- No  Others- Yes | IDA- Single  Other- Double | Small | Impact | Medium | Small- not powered | Medium |
| Lozoff (2003) [50] | Yes | Formula- low dose iron | Formula- ad libitum | Good | Yes- but differences remained | Double | Medium | Impact | No effect | Large- not powered | Medium |
| Lozoff (2010) [48] | Yes | Poor- before and after | Good | Poor- long term relevance? | No | No | Small | Impact | Large | Small- not powered | High |
| Morley (1999) [70] | Not reported- likely low | Formula- low dose iron | Formula- ad libitum | Good | Yes | Double | Large-lost group were lower SES | Some impact | No effect | Large- powered for medium effect | Medium |
| Oski (1978) [67]  Honig (1978) [77] | Yes | Good | Fair- wide age range | Poor- 1 week after baseline | Yes | Double | Small | No report | Large | Small- not powered | High |
| Oski (1983) [76] | Yes | Good | Fair- wide age range | Poor- 1 week after baseline | Yes | Double | Small | No report | Large | Small- not powered | High |
| Sarici (2001) [46] | Yes | Poor | Fair- wide age range | Fair | No | No | Small | Impact | No effect | Small- not powered | High |
| Walter (1983) [75] | Yes | Poor- Before and after | Poor- very short | Good | No | No | Large | Not reported | IDA improved in cognitive, and socio-emotional | Small- not powered | High |
| Walter (1989) [32] | Yes | Poor- all received iron after initial 10 days. | Good- partial observation | Good | Yes for initial 10 days | Double | Small | Impact | No effect | Medium- not powered | High |
| **Supplementation from 2-4 years** | | | | | | | | | | | |
| **Author (Year)** | **Iron Deficiency Prevalent?** | **Utility of Control Group** | **Utility of Intervention** | **Utility of outcome** | **Randomised/ Groups differences** | **Blinded?** | **Loss to follow up** | **Treatment effect (iron)** | **Treatment effect (brain)** | **Sample Size** | **Overall Risk of Bias.** |
| Kabakus (2002) [49] | Not stated | Poor- before and after | Fair- wide age range | Poor- long term relevance? | No | No | Small | Impact | Unclear | Small- not powered | High |
| Metallinos-Kataras (2004) [78] | Yes | Good | Fair- maybe micronutrient interactions but admin at day care | Fair- Not standardised test | Yes | Double | Large | Impact | Inconsistent effect | Small- not powered | High |
